# Rapid and Facile Synthesis of High-Performance Silver Nanowires by a Halide-Mediated, Modified Polyol Method for Transparent Conductive Films

**DOI:** 10.3390/nano10061139

**Published:** 2020-06-09

**Authors:** Lin Cao, Qin Huang, Jie Cui, Huaijun Lin, Wei Li, Zhidan Lin, Peng Zhang

**Affiliations:** 1 Institute of Advanced Wear & Corrosion Resistant and Functional Materials, Jinan University, Guangzhou 510632, China; linc19993@163.com (L.C.); hq2502@126.com (Q.H.); hjlin@jnu.edu.cn (H.L.); liweijnu@126.com (W.L.); 2 Analytical and Testing Center, South China University of Technology, Guangzhou 510640, China; czcuijie@scut.edu.cn

**Keywords:** silver nanowires, modified polyol method, rapid preparation, halides, transparent conductive films

## Abstract

Silver nanowires are receiving increasing attention as a kind of prospective transparent and conductive material. Here, we successfully synthesized high-performance silver nanowires with a significantly decreased reaction time by a modified polyol method. The synthesis process involved the addition of halides, including NaCl and NaBr, to control the release rate of Ag^+^ ions, as Cl^−^ and Br^−^ ions react with Ag^+^ ions to form AgCl and AgBr with different solubilities. As a result, Ag^+^ ions could be slowly released by graded dissolution, and the formation of silver nanowires was promoted. The results showed that the concentration of the added halides played an important role in the morphology of the final product. High-quality silver nanowires with an average diameter of 70 nm and average length of 21 μm were obtained by optimizing the reaction parameters. Afterwards, a simple silver nanowire coating was applied in order to fabricate the transparent conductive films. The film that was based on the silver nanowires provided a transmittance of 91.2% at the 550 nm light wavelength and a sheet resistance of about 78.5 Ω·sq^−1^, which is promising for applications in flexible and transparent optoelectronic devices.

## 1. Introduction

Transparent conductive materials have been recognized as being important in optoelectronic devices, such as liquid crystal displays (LCDs), touch panels, organic light-emitting diodes (OLEDs), and solar cells [1,2]. Indium tin oxide (ITO) has been the most widely used transparent conductive material, which is composed of approximately 90% In_2_O_3_ and 10% SnO_2_. ITO films exhibit a low sheet resistance and relatively high transmittance in the visible light region. However, they are limited in flexible electronic devices due to their mechanical brittleness, expensive vacuum deposition process, and the rarity of indium [3,4]. Therefore, potential alternative transparent conductive materials are being sought to substitute ITO. Several alternative materials have attracted considerable research interest, including carbon-based materials (graphene [5,6,7,8] and carbon nanotubes [9,10,11,12]), poly(3,4-ethylenedioxythiophene):poly(styrenesulfonate) (PEDOT:PSS) [13,14] in conductive polymers, and metal nanomaterials, such as silver nanowires [15,16,17] and copper nanowires [18,19,20]. Among these alternatives, the electrical conductivity and transmittance of carbon-based materials are inferior to silver nanowires [21]. Although conductive polymers can be easily prepared for a transparent conductive film, the conductivity is also relatively poor [22]. Above all, silver nanowires have been considered to be an especially promising candidate for an ITO substitute. In comparison to the ITO films, silver nanowire conductive films prepared on flexible substrates can provide high flexibility as well as high light transmittance and electrical conductivity. Therefore, it would be easily incorporated into low-resistance and high-transmittance portable optoelectronic devices [23].

The transmittance and sheet resistance of transparent conductive films that are based on silver nanowires are strongly related to the diameter and length of the nanowires. Generally, silver nanowires with a smaller diameter and longer length exhibit a higher transmittance and lower sheet resistance, which are crucial factors for applications in transparent electrodes. A sparse network with a low number of links formed by longer silver nanowires can provide more “open areas”, thereby helping to increase the transmittance [24]. Meanwhile, fewer percolation network connections can be generated, which would assist in decreasing junction resistance [25,26]. Furthermore, the ability to resist mechanical deformation is extremely different, which is, a dense network is unable to withstand large strains, but a sparse network that is connected by a lower number of long silver nanowires can adapt to mechanical deformations effectively and prevent cracks from generating [27].

Although a variety of silver nanowire synthesis methods have been proposed, a simple and rapid synthetic strategy is still needed to advance the field. The Polyol method is widely adopted, because of its low cost, mild reaction conditions, and its suitability for industrial production [28]. Wiley introduced Cl^−^ and Br^−^ into the polyol reaction, which was heated under nitrogen for nearly 2 h, and silver nanowires with a diameter of 20 nm were acquired [29]. Recently, Wei added NaCl under a reaction temperature of 160 °C, and the obtained silver nanowires were 50~90 nm in diameter and 15~25 μm in length [30]. Silver nanowires with different aspect ratios were prepared while ignoring the complexity of the synthesis process and long reaction time [29,30,31,32,33,34,35]. Thus, it is of great significance to develop a silver nanowire synthesis strategy that is fast, simple, and cost-effective.

In the present work, we adopted a modified polyol method with two halides to assist in the synthesis of silver nanowires with high aspect ratios, which greatly decreased the reaction time when compared to previously reported research with the polyol method. In the work of Tang et al. [32], uniform silver nanowires were synthesized via a green and simple hydrothermal method through fine tailoring the anions in the precursors. Additionally, the reaction time is 18 h. Rui et al. [36] investigated the roles of Cl^−^ and Br^−^ in the preparation of AgNWs and then synthesized high aspect ratio (up to 2100) AgNWs in high yield (>85% AgNWs) using a Cl^−^ and Br^−^ co-mediated method in 30 min. Tetsumoto et al. [37] found that, in the AgNO_3_, NaCl and lucose system, the AgNWs could be easily generated at temperature of 150 °C. Additionally, a necessary reaction time of 24 h was required to guarantee the gradual transformation from Ag to AgNWs.

In this method, ethylene glycol (EG) was used as a polyol to achieve the dual roles of being a reducing agent and solvent, and NaCl and NaBr were used as control agents to combine with Ag^+^ ions to form insoluble AgCl and AgBr. Uniform silver nanowires were obtained through the slow release of Ag^+^ ions and the fast growth rate of the (111) plane. Furthermore, the growth mechanism and the effects of various reaction parameters on the morphology of silver nanowires were discussed. The transparent conductive film that was fabricated with the as-synthesized silver nanowires exhibited a good performance, demonstrating its potential application in optoelectronic devices.

## 2. Materials and Methods

### 2.1. Materials

Silver nitrate (AgNO_3_, 98%) was acquired from Sinopharm Chemical Reagent Co., Ltd. (Shanghai, China), sodium chloride (NaCl, 99.9%) was obtained from Sigma–Aldrich (St. Louis, MO, USA)., sodium bromide (NaBr, 99.0%) was provided by Fuchen Chemical Reagent Factory (Tianjin, China), polyvinylpyrrolidone (PVP, MW ≈ 1300000) was supplied by Macklin Biochemical Co., Ltd. (Shanghai, China), ethylene glycol (EG, 99.8%), and absolute ethanol (99.7%) were acquired from Damao Chemical Reagent Factory (Tianjin, China). All of the chemicals were applied without further purification.

### 2.2. Synthesis of Silver Nanowire

In a typical synthesis process, 0.01 M NaCl and 0.005 M NaBr dissolved in EG were prepared in advance. Subsequently, 5.2 mmol of PVP was dissolved in 19 mL of EG under stirring. Following this, 0.6 mL of NaCl solution and 1.6 mL of NaBr solution, 3.5 mmol of AgNO_3_ was added in sequence and mixed well for 1 min. The mixed solution was placed in a preheated 175 °C oil bath for being heated about 16~18 min. It is worth mentioning that the magnetic stirring was employed throughout the synthesis process in order to maintain a uniform solution, and all of the reagents were poured into at once and did not need to be slowly introduced in a dropwise or stepwise manner for the reaction. Finally, the reaction mixture was diluted with ethanol purified to remove excess reagent after the reaction finished and centrifuged at 8000 rpm for 5 min., the centrifugation process was repeated several times until the supernatant became colorless. The collected silver nanowires were redispersed in ethanol for further use and characterization.

### 2.3. Preparation of Silver Nanowires Transparent Conductive Films

A flexible transparent conductive film that was based on polyethylene terephthalate (PET) was fabricated. The PET films were ultrasonically washed in detergent, deionized water, and ethanol for 10 min., respectively. The as-prepared silver nanowires that were dispersed in absolute ethanol were coated on the PET substrate and uniformly coated by a bar coating manner. Different silver contents films were prepared for investigating the effects of silver amounts on the optoelectronic performance.

### 2.4. Characterization of Silver Nanowires and Transparent Conductive Films

The surface morphology of the silver nanowires was observed by field emission scanning electron microscopy (SEM, ULTRA 55, Zeiss, Oberkochen, Germany). The phase composition and structure were analyzed by X-ray diffraction (XRD, D8 Advance, Bruker, Berlin, Germany) at a scan rate of 2°/min with Cu Kα (λ = 1.54056 Å) radiation. Transmission electron microscopy (TEM, JEM-2100F, JEOL Ltd., Tokyo, Japan) was employed to analyze the crystal structure. The UV-visible spectrophotometer (UV-2550, Shimadzu Corporation, Tokyo, Japan) was used to characterize the UV-visible absorption spectrum of silver nanowires and the optical transmittance of transparent conductive films. The sheet resistance of the conductive films were carried out by a four-point probe method (KDB-1, Guangzhou Kunde Technology Co., Ltd., Guangzhou, China). Silver nitrate (AgNO_3_, 98%) was acquired from Sinopharm Chemical Reagent Co., Ltd., sodium chloride (NaCl, 99.9%) was obtained from Sigma–Aldrich (St. Louis, MO, USA)., sodium bromide (NaBr, 99.0%) was provided by Fuchen Chemical Reagent Factory (Tianjin, China), polyvinylpyrrolidone (PVP, MW ≈ 1,300,000) was supplied by Macklin Biochemical Co., Ltd. (Shanghai, China), ethylene glycol (EG, 99.8%), and absolute ethanol (99.7%) were acquired from Damao Chemical Reagent Factory (Tianjin, China).

## 3. Results

### 3.1. Characterization of Silver Nanowires

**Figure 1:**
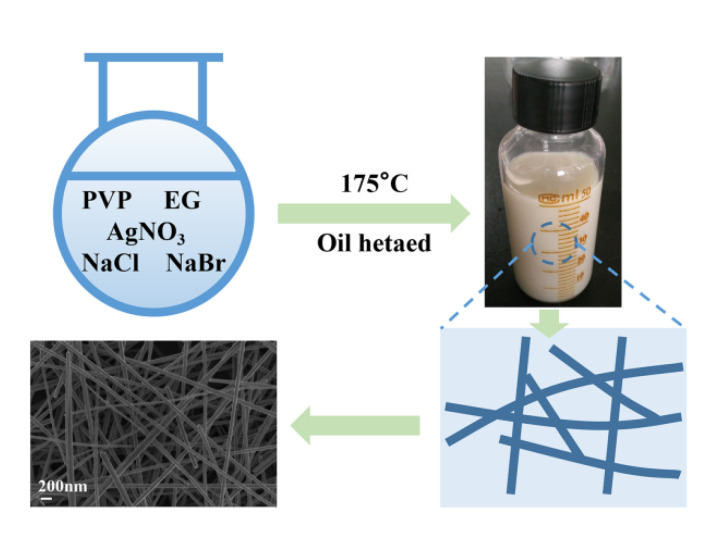
Synthesis of silver nanowires.

Figure 1 illustrates the synthesis of silver nanowires. Silver nanowires were prepared by the NaCl- and NaBr-mediated modified polyol method. All of the reactants were sequentially added to form a homogeneous reaction solution, and the reaction was carried out under high temperature. Initially, the EG in the reaction solution was dehydrated to acetaldehyde, and the Ag^+^ ions were reduced to Ag^0^. At the same time, Ag^+^ ions in the solution combined with Cl^−^ and Br^−^ ions to form insoluble AgCl and AgBr in order to control the formation of silver nanowires. Subsequently, after the completion of the reaction, silver nanowires were obtained after centrifugal purification and were dispersed in absolute ethanol solution. The possible reaction mechanism is presented in the following equations.

(1) $$2OHCH_{2}-CH_{2}OH\to 2CH_{3}CHO+2H_{2}O$$

(2) $$2Ag^{+}+2CH_{3}CHO\to 2Ag+2H^{{}_{+}}+CH_{3}CO-COCH_{3}$$

(3) $$Ag^{+}+Cl^{-}\Leftrightarrow AgCl$$

(4) $$Ag^{+}+Br^{-}\Leftrightarrow AgBr$$

**Figure 2:**
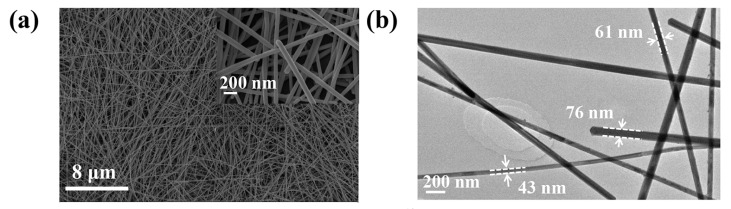
(**a**) Typical scanning electron microscopy (SEM) image (inset shows the corresponding high magnification) and (**b**) transmission electron microscopy (TEM) image of silver nanowires.

The surface morphology of the as-prepared silver nanowires was observed by SEM and TEM. The silver nanowires exhibited long and uniform one-dimensional shapes, with diameters of about 40–80 nm, which clearly showed that the obtained silver nanowires were well-defined, as shown in Figure 2a,b. Figure S1 shows the SEM image of silver nanowires at a low magnification, in which a length over 40 μm and high flexibility can be clearly seen. It can be seen from the figures that the length of the as-synthesized silver nanowires ranged from 8 to 38 μm, and the diameter ranged from 30 to 170 nm. It is worth mentioning that, although the distribution of length was relatively large, most of the nanowires were below 100 nm. An average length of about 21 μm and a diameter of 70 nm were obtained by calculation. These results indicate that the modified polyol method can successfully synthesize silver nanowires with good morphology in a short time.

**Figure 3:**
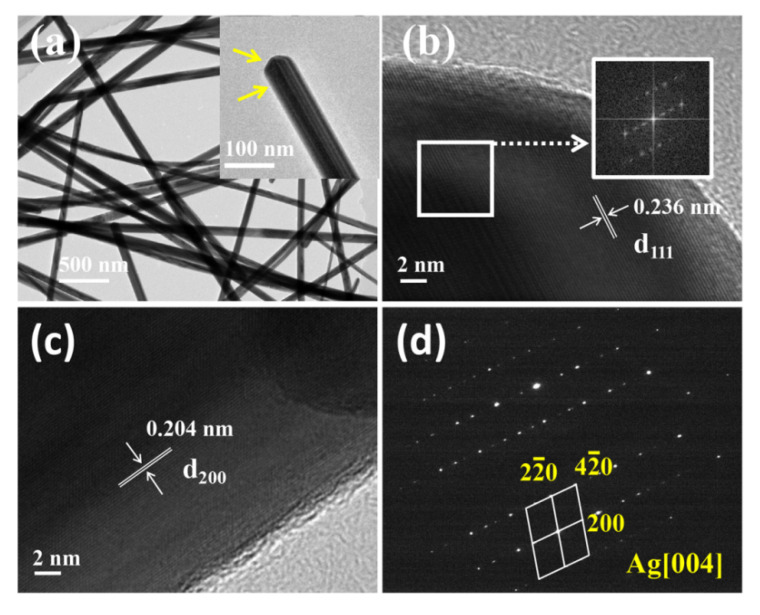
(**a**) Detailed TEM image of single silver nanowires, (**b**) and (**c**) High-resolution transmission electron microscopy (HRTEM) images of the tip and side regions of a single silver nanowire, respectively, and (**d**) typical selected area electron diffraction (SAED) pattern of the tip region.

Further analysis was undertaken for silver nanowires by TEM. The TEM image (Figure 3a) clearly shows the detailed morphology of a single silver nanowire, which exhibited a sharp end face and the pentagonal end face that comprised five (111) planes [38]. The tip regions and the side regions were clearly observed, as displayed in the high-resolution TEM images (Figure 3b,c). The lattice spacings were 0.236 nm and 0.204 nm, respectively, which was in good agreement with the d value of the (111) plane and (100) plane of FCC Ag. The inset in Figure 3b shows the fast Fourier transform pattern, suggesting a twinned crystallographic orientation structure of silver nanowires. The selected area electron diffraction (SAED) image (Figure 3d) that was obtained by focusing the electron beam onto the (111) plane of the tip region showed a plurality of electron diffraction patterns and diffraction spots, which indicates that the as-synthesized silver nanowires had a multiple twin structure, which is consistent with a previous report [39].

**Figure 4:**
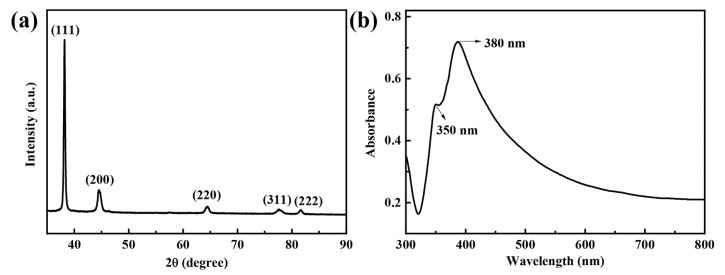
(**a**) X-ray diffraction (XRD) pattern and (**b**) UV–vis absorption spectrum of silver nanowires.

The XRD pattern in Figure 4a was further used to demonstrate the structure and crystallinity of silver nanowires. The diffraction peaks of the (111), (200), (220), (311), and (222) planes can be assigned to the face-centered cubic (FCC) structure of silver (JCPDS File No. 04-0783). The diffraction intensity ratios of the (111)/(200) planes and the (111)/(220) planes were 6.5 and 19.6, respectively, while the standard PDF cards were 2.5 and 4, respectively, demonstrating that there was preferential growth in (111) plane. PVP promotes the addition of silver atoms to the (111) crystalline plane (both ends of the silver nanowires) by stronger adsorption with the (100) side of the silver (the side of the silver nanowires). In other words, most of the PVP was coated on the (100) crystalline plane, and the exposed (111) crystalline plane grew naturally faster than the other crystal planes. At the same time, the halogen ions in the solution could form a halogenated salt with the silver ions, which played the role of slowly releasing Ag^+^ ions. Finally, it continuously grew into silver nanowires. Figure 4b illustrates different shapes of silver nanostructures would exhibit different surface plasmon resonance peaks. Furthermore, the UV–visible absorption spectrum of silver nanowires [40]. Two characteristic absorption peaks of silver nanowires at ~350 nm and ~380 nm can be observed. The shoulder at 380 nm is attributed to the quadrupole resonance excitation, and the main peak at 350 nm belongs to the lateral plasmon resonance of the silver nanowires. The result of the UV–visible absorption spectrum further proved that silver nanowires could be obtained by the method.

### 3.2. Growth Mechanism of Silver Nanowires

**Figure 5:**
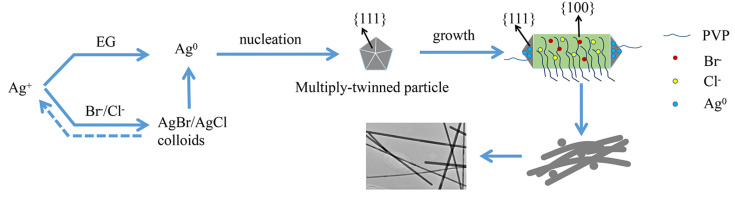
Schematic illustration of the growth mechanism of silver nanowires in a modified polyol synthesis process.

Figure 5 illustrates the growth mechanism of the silver nanowires. During the formation of silver nanowires, initially a portion of Ag^+^ ions would be directly reduced to Ag^0^ by EG. Subsequently, the aggregated Ag^0^ gradually grew into a pentagonal multiple-twinned silver seed and further generated silver nanorods, followed by the final formation of silver nanowires. It is worth pointing out that Ag^0^ would also inevitably generate some single crystals or single twins while growing into the multiple-twinned silver seed. At the same time, the presence of Cl^−^ and Br^−^ ions also imparts a degree of oxidative etching to multiple-twinned particles, since Cl^−^ or Br^−^/O_2_ could more easily oxidize multiple-twinned particles [41]. However, the Br^−^ ion-capping agent could be adsorbed on the (100) crystal plane of silver by virtue of its smaller size more easily than the polymer-capping agent PVP. The addition of Br^−^ and Cl^−^ ions could greatly slow down the release rate of Ag^+^ ions in the solution; thus, the reduction rate decelerated, leading to Ag^0^ being slowly added to the multiple-twinned particles. The decrease in the diameter of silver nanowires favors their formation at a high aspect ratio [42]. Namely, the decreased reduction process kinetics of the Ag^+^ ions would prevent the Ag^+^ ions concentration from being too high, and a large amount of Ag^0^ would be generated under EG reduction, resulting in the formation of silver nanoparticles. In other words, a portion of Ag^+^ ions would combine with Cl^−^ and Br^−^ ions, and AgCl/AgBr colloids would be formed. The generated insoluble amorphous silver halide could be reduced by EG after dissolving Ag^+^ ions. In the meantime, Ag^0^ could be directly produced under the reduction of EG. The reduced Ag^0^ might participate in the formation of initial multiple-twined particles or be added to the already formed silver nanowires.

In addition to slowing down the release rate of Ag^+^ ions, the introduction of NaBr and NaCl played other key roles in the formation of silver nanowires. The silver nanowires consisted of ten (111) crystalline planes and five (100) crystalline planes. In terms of surface free energy, the (100) plane was larger than the (111) plane. Thereby, the (100) plane could more strongly bind to PVP and Br^−^ and Cl^−^ ions that are present in the reaction mixture due to the higher surface free energy, and this resulted in passivation of the (100) plane, inhibiting the growth rate of the lateral (100) plane and promoting the tendency of the Ag^0^ to be deposited onto the (111) plane. Additionally, Cl^−^ ions could stabilize the initial formation process and prevent the aggregation of the multi-twinned silver seeds [43], which acted as an electrostatic stabilizer. Moreover, the solubility product constant of AgCl (Ksp = 1.56 × 10^−10^) was larger than that of AgBr (Ksp = 7.7 × 10^−13^). In this case, the AgBr precipitate preferentially formed in the solution, but the standard reduction potential of AgBr (0.071 V) was lower than that of AgCl (0.2223 V), causing the reduction rate of AgCl to be greater than that of AgBr. Thus, Ag^+^ ions could be released by graded dissolution, which is effective in controlling the Ag^+^ ions concentration over the reduction reaction. In addition, the PVP molecular chain allows the silver nanowires to be better dispersed in the solution, thus reducing the occurrence of agglomeration. In a word, the newly formed silver atoms are preferentially deposited onto the (111) plane of the silver, causing the (111) plane to grow faster than the other crystalline planes and effectively avoiding the lateral growth of the silver nanowires. As a result, silver nanowires with a high aspect ratio can be generated through anisotropic growth [44].

### 3.3. Influence of the Reaction Parameters

**Figure 6:**
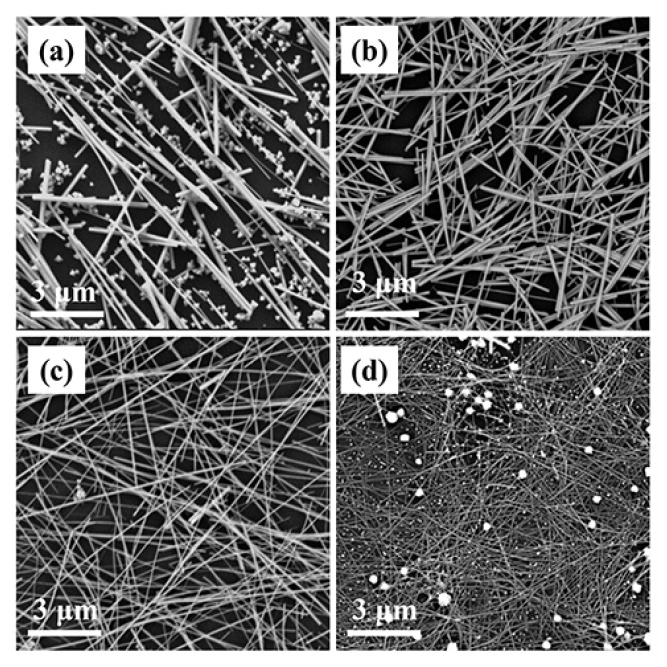
SEM images of silver nanowires synthesized with different concentrations of NaBr: (**a**) 0 mM, (**b**) 0.185 mM, (**c**) 0.370 mM, and (**d**) 0.74 mM.

Different reaction conditions have different effects on the morphology of products. Here, the effects of the concentrations of Br^−^ and Cl^−^ ions, reaction temperature, reaction time, and the molecular weight of PVP on the formation of well-defined silver nanowires were investigated. The effect of Br^−^ ions on the silver nanowires morphology was investigated by changing the concentration of NaBr. Figure 6 shows the SEM images of the synthesized products in different NaBr concentrations, while the other reaction conditions were unchanged. When no Br^−^ ions were added, the final product was a mixture of micrometer rods and nanoparticles (Figure 6a), whereas coarser nanorods became the main product at an NaBr concentration of 0.185 mM (Figure 6b). As the NaBr concentration increased to 0.370 mM, the diameter of the silver nanowires decreased noticeably (Figure 6c). However, upon increasing the concentration to 0.740 mM, the number of irregular particles increased obviously, though silver nanowires became thinner (Figure 6d). This might be due to the rate of nucleation being increased at high Br^−^ ion concentrations, causing not enough Ag^0^ to be produced for polycrystalline particles to grow into silver nanowires, which suggests that Br^−^ ions play a significant role in the morphology of the final product. Similarly, Cl^−^ ions also played an important role in the synthesis of silver nanowires. When only NaBr was introduced as the control agent, the products were particles, as shown in Figure S2. The products did not become nanowires until the concentration of NaCl increased to 0.283 mM. However, as the concentration of NaCl rose to 0.472 mM, silver nanoparticles in the products increased again. This might be due to the consumption of more Ag^+^ ions at a higher concentration that combined with Cl^−^ ions, and AgCl colloids formed, resulting in less Ag^+^ ions being reduced in time to Ag^0^ required for growth.

In addition to the concentration of Br^−^ and Cl^−^ ions, the reaction temperature and reaction time also had important influences on the formation of silver nanowires. The other reaction parameters were kept constant, and silver nanowires were synthesized at three different reaction temperatures, in order to investigate the effect of reaction temperature on the morphology of silver nanowires, as shown in Figure S3. For a reaction temperature of 165 °C, the product contained a large number of silver nanoparticles and a small number of silver nanowires and nanorods (Figure S3a). As the reaction temperature increased to 175 °C, the majority of the products were silver nanowires (Figure S3b). However, when the reaction temperature continued to increase to 185 °C, the silver nanowires in the products became significantly shorter than those that were synthesized at 175 °C (Figure S3c). In addition, some nanorods and large particles were also included. It can be seen from these results that the reaction temperature had a strong influence on the formation of silver nanowires. On the one hand, the nucleation rate and the growth rate both increased as the reaction temperature increased. On the other hand, the reaction temperature affected the reducing ability of EG. When the temperature was relatively low, the crystal nucleus formed initially at a small nucleation growth rate and lacked sufficient energy to form silver nanowires. Generally, the reducing ability of the system accelerated with the increase of reaction temperature. Meanwhile, the kinetic diffusion mass transfer conditions were greatly improved, which promoted the growth of silver nanowires. As the reaction temperature continued to increase, the reducing ability advanced greatly, the probability of crystal nucleus collision and agglomeration increased, and the growth rate was quicker than the nucleation rate. Therefore, shorter silver nanowires were formed.

**Figure 7:**
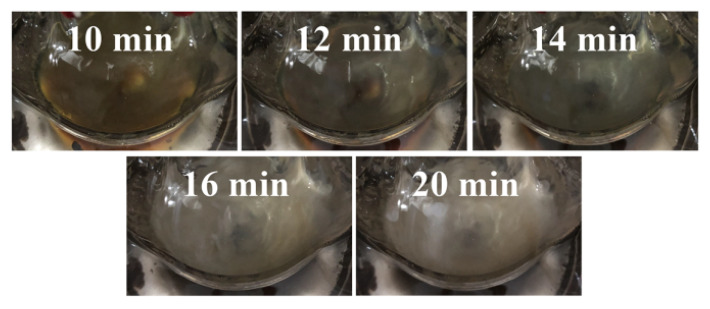
Digital photos of silver nanowires in different stages of growth.

**Figure 8:**
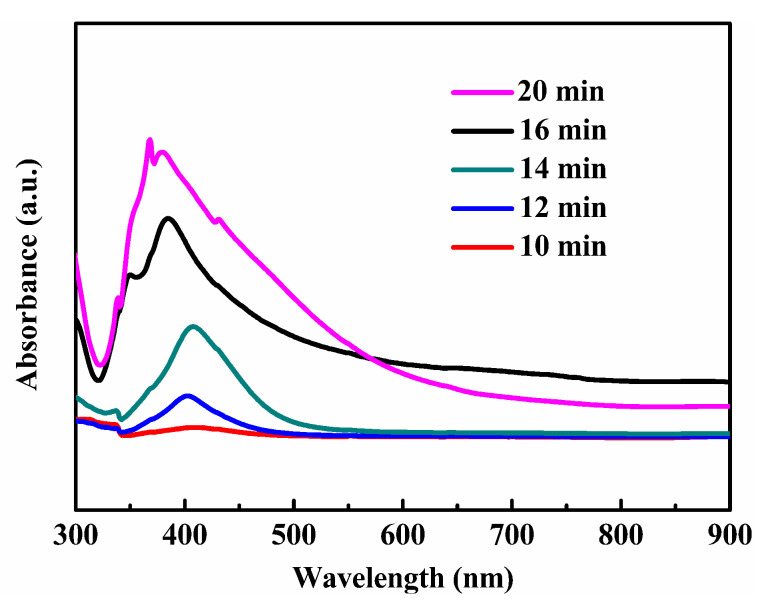
UV–vis absorption spectra of the silver nanomaterials at different reaction stages.

Figure 7 shows the digital photos in different growth stages. Evidently, the reaction time had an important influence on the growth of silver nanowires. The color of the solution varies with the reaction time. The solution began to turn brown when the reaction was carried out for 10 min., which indicated that silver nanoparticles began to form in the solution. As the reaction continued, the solution began to become turbid and, when the reaction lasted for 16 min., the solution turned gray and white, at which point the reaction ended. If the reaction continues, the color of the solution does not change much, which indicated that there is no longer any chemical change and the reaction is complete. The changes in the silver nanowire synthesis process were detected by a UV–vis spectrophotometer. Figure 8 shows that the silver nanostructure varied greatly in different reaction stages. When the reaction time was 10 min., silver nanoparticles began to form in the solution. As time continued to increase to 12 min, the clear absorption peak at 410 nm represented silver nanoparticles. When the reaction reached 14 min., the absorption peak appeared red-shifted, indicating the growth of silver nanorods. Further raising the time to 16 min., the absorption peak of silver nanowires appeared at about 35 and 380 nm. Upon further growth to 20 min., the absorption peak of the silver nanowire at 350 nm disappeared, and the silver nanorod was excessively grown.

The effect of molecular weight of PVP on the morphology of silver nanowires was further investigated. Figure S4 shows the SEM images of synthesized products with different PVP molecular weights. The silver particles aggregated to form a cluster in the case of a molecular weight of 8000 (Figure S4a). As the molecular weight of PVP increased to 24,000, a mixture of nanorods and nanoparticles formed (Figure S4b). Short silver nanowires were formed for a molecular weight of 58,000 (Figure S4c). With the molecular weight increasing to 1,300,000, silver nanowires with better quality and higher yield could be seen in Figure S4d. It can be seen that the difference in molecular weights of PVP also had different performances for synthetic silver nanowires. This is possible because preferential adsorption is difficult under the circumstances that the binding force to Ag^0^ is relatively small, as the PVP with a low molecular weight has a smaller viscosity than PVP with a high molecular weight. As a consequence, silver nanoparticles tend to form. As the molecular weight of PVP continued to increase, the viscosity of the reaction system increased, and the characteristic adsorption was enhanced. Typically, PVP would preferentially bind to the (100) plane, causing the (111) plane to grow at a rate faster than the (100) plane, thereby growing into a one-dimensional silver nanowire.

### 3.4. Performance of Silver Nanowire Transparent Conductive Films

**Figure 9:**
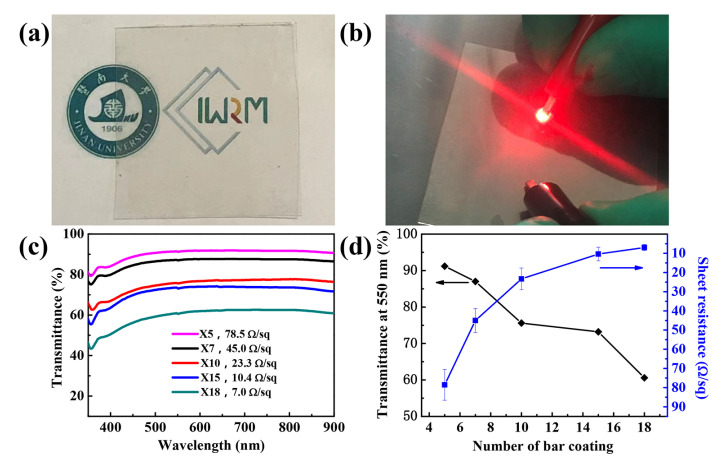
(**a**) Fabricated silver nanowire transparent conductive film on PET, (**b**) test of conductivity performance, (**c**) plot of transmittance versus wavelength of as-fabricated silver nanowire transparent conductive films under different bar coating times, and (**d**) transmittance and sheet resistance at different bar times of as-fabricated silver nanowire transparent conductive films.

The as-synthesized silver nanowires were dispersed in absolute ethanol to prepare a silver nanowire suspension of approximately 5 mg·mL^−1^. Subsequently, it was applied to the surface of the PET substrate by bar coating. Transparent conductive films with different performances could be obtained by changing the number bar coatings. It can be clearly seen that the fabricated film was transparent, as shown in Figure 9a. Additionally, the illuminated light-emitting diodes (LEDs) indicated that the film was electrically conductive (Figure 9b). The sheet resistance and transmittance of the films were further studied. When the bar coating was applied five times (expressed as ×5), the obtained conductive films provided a transmittance of 91.2% at a wavelength of 550 nm, and the average sheet resistance was about 78.5 Ω·sq^−1^ (Figure 9c). This is promising for replacing conventional ITO conductive films. As is expected, as the number of bar coatings increased, the sheet resistance showed a tendency to decline. The transparent conductive films required both a low sheet resistance and a high transmittance. Figure 9d intuitively shows the relationship between the number of bar coatings and the transmittance, as well as the sheet resistance. It can be clearly seen that, as the number of bar coatings increased, the transmittance of the films decreased. However, the sheet resistance showed an upward trend. This means that, if a transparent conductive film has a high transmittance, it inevitably sacrifices a certain conductivity. Therefore, it is essential to find an appropriate point between the required transmittance and conductivity, so that a transparent conductive film can be obtained that satisfies the demand.

## 4. Conclusions

In conclusion, well-defined silver nanowires with an average diameter of 70 nm and average length of 21 μm were successfully prepared. In particular, the reaction time decreased remarkably; thus, it demonstrated improved productivity. The yield and quality of silver nanowires were closely related to reaction temperature, PVP molecular weight, and halide concentration. The introduction of Br^−^ and Cl^−^ ions could decrease the release rate of Ag^+^ ions by graded dissolution and promote the formation of high-quality silver nanowires. Silver nanowires with high aspect ratios were obtained with optimized reaction conditions. As for the fabricated silver nanowire transparent conductive film, it exhibited a transmittance of 91.2% at the wavelength of 550 nm and a sheet resistance of 78.5 Ω·sq^−1^. The results manifest the wide potential application prospects of transparent conductive films that are based on silver nanowires in flexible and transparent optoelectronic devices.

## Supplementary Materials

The following are available online at https://www.mdpi.com/2079-4991/10/6/1139/s1, Figure S1: SEM image of the as-synthesized silver nanowires at x3000 magnification, Figure S2: SEM images of silver nanowires synthesized with different concentrations of NaCl: (**a**) 0 mM, (**b**) 0.189 mM, (**c**) 0.283 mM, and (**d**) 0.472 mM, Figure S3: SEM images of silver nanowires synthesized at different reaction temperature: (**a**) 165 °C, (**b**) 175 °C, and (**c**) 185 °C, Figure S4: SEM images of silver nanowires synthesized with different molecular weights of PVP: (**a**) 8,000, (**b**) 24,000, (**c**) 58,000, (**d**) 1,300,000.
