# Supplementary material for: Rapid and Facile Synthesis of High-Performance Silver Nanowires by a Halide-Mediated, Modified Polyol Method for Transparent Conductive Films
Source: Nanomaterials (Basel). 2020 Jun 9;10(6):1139. doi: 10.3390/nano10061139 (PMC7353346; doi:10.3390/nano10061139)
Supplement: Supplementary file 1 [file nanomaterials-10-01139-s001.pdf]

nanomaterials

Supplementary Materials

Rapid and Facile Synthesis of High-Performance Silver Nanowires by A Halide-Mediated, Modified Polyol Method for Transparent Conductive Films

Lin Cao [1], Qin Huang [1], Jie Cui [2], Huaijun Lin [1], Wei Li [1], Zhidan Lin [1,*], and Peng Zhang [1,*]

[1] Institute of Advanced Wear & Corrosion Resistant and Functional Materials, Jinan University, Guangzhou 510632, China; linc19993@163.com (L.C.); hq2502@126.com (Q.H.); hjlin@jnu.edu.cn (H.L.); liweijnu@126.com (W.L.)
[2] Analytical and Testing Center, South China University of Technology, Guangzhou 510640, China; czcuijie@scut.edu.cn
* Correspondence: linzd@jnu.edu.cn (Z.L.); tzhangpeng@jnu.edu.cn (P.Z.); Tel.: +86-020-8522-3562 (P.Z.); Fax: +86-020-8522-0890 (Z.L.)

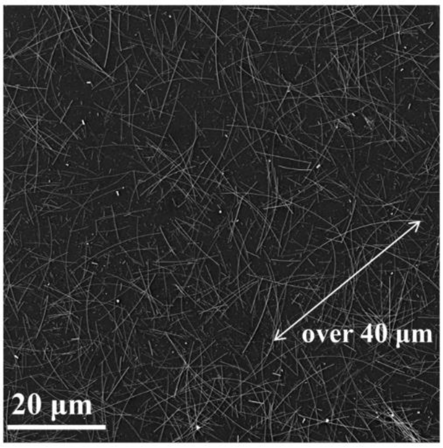

Figure S1. SEM image of the as-synthesized silver nanowires at x3000 magnification.

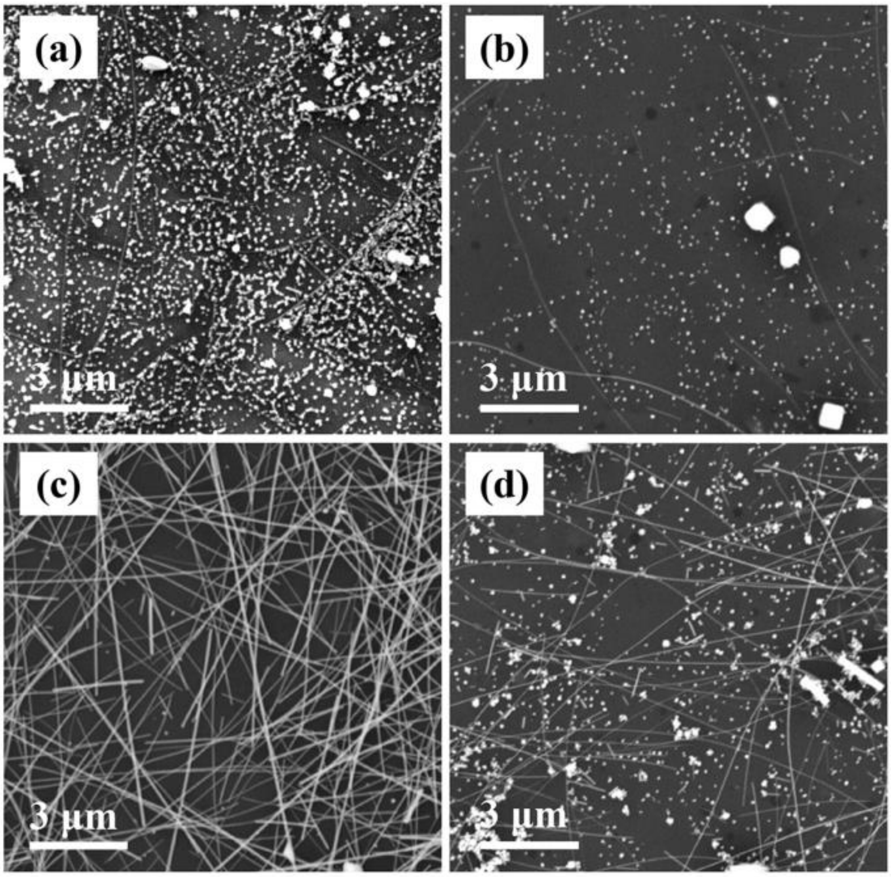

Figure S2. SEM images of silver nanowires synthesized with different concentrations of NaCl: (**a**) 0 mM, (**b**) 0.189 mM, (**c**) 0.283 mM, and (**d**) 0.472 mM.

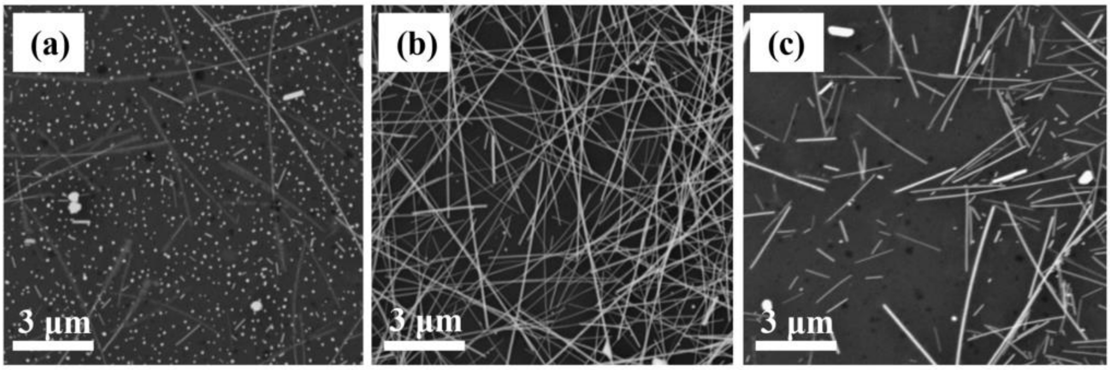

Figure S3. SEM images of silver nanowires synthesized at different reaction temperature: (**a**) 165 °C, (**b**) 175 °C, and (**c**) 185 °C.

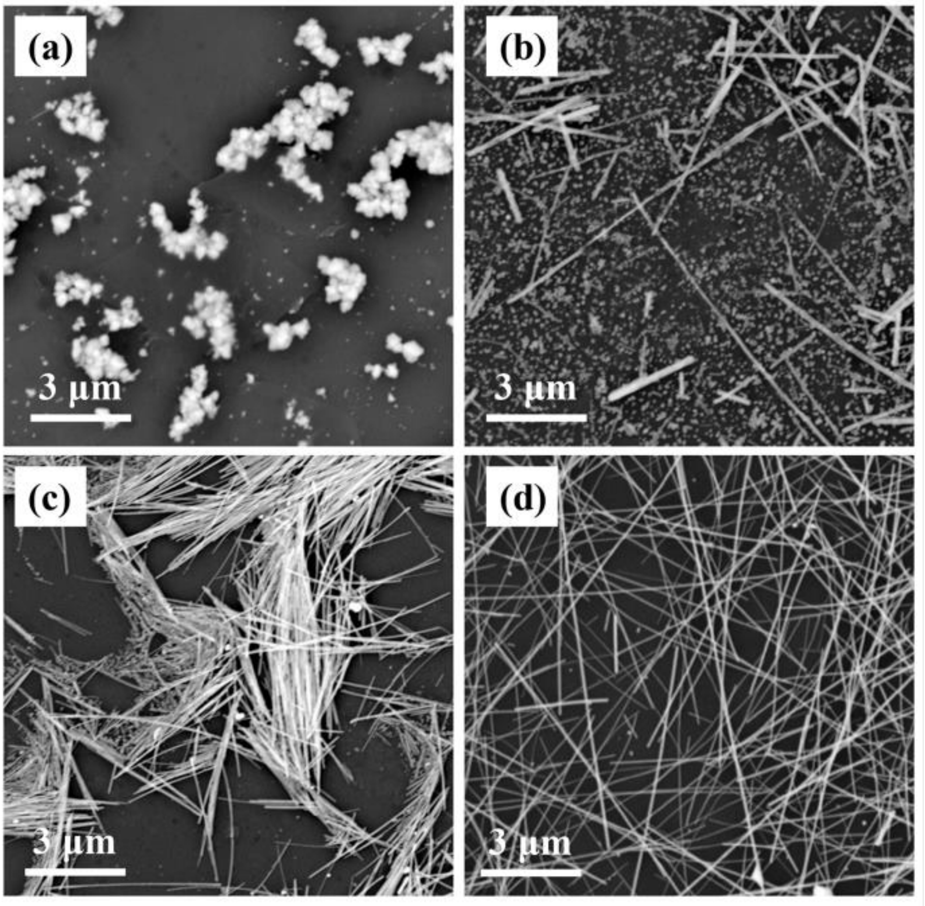

Figure S4. SEM images of silver nanowires synthesized with different molecular weights of PVP: (**a**) 8,000, (**b**) 24,000, (**c**) 58,000, (**d**) 1,300,000.
